# Association between Liver Fluke Infection and Hepatobiliary Pathological Changes: A Systematic Review and Meta-Analysis

**DOI:** 10.1371/journal.pone.0132673

**Published:** 2015-07-17

**Authors:** Jing Xia, Shi-chen Jiang, Hong-Juan Peng

**Affiliations:** Department of Pathogen Biology, Guangdong Provincial Key Laboratory of Tropical Disease Research, School of Public Health and Tropical Medicine, Southern Medical University, Guangzhou, Guangdong, the People’s Republic of China; Hebrew University, ISRAEL

## Abstract

**Objective:**

To provide information about the role of liver fluke infection as a risk factor for hepatobiliary pathological changes and promote awareness among the people living in endemic areas, a systematic review and meta-analysis based on published studies was conducted to examine the association between liver fluke infection and hepatobiliary pathological changes.

**Methods:**

Relevant original literature was searched in multiple literature databases, including PubMed, Cochrane, Clinical Evidence, Trip Database, Clinical Trials, Current Controlled Trials, Web of Science, the China National Knowledge Infrastructure (CNKI) database, and the Wanfang academic journal full-text database. Studies were selected based on strict screening with inclusion and exclusion criteria. Tests of heterogeneity, sensitivity and publication bias were performed with the Review Manager software, version 5.3, and meta-regression analyses were performed with the Stata software, version 11.0 (Stata Corporation, College Station, TX, USA). Pooled risk ratios (RRs) and odds ratios (ORs) with their 95% confidence intervals (95% CIs) were calculated and used to evaluate the risk of hepatobiliary pathological changes resulting from liver fluke infection. Linear trend analyses were conducted to determine the dose-response relationship using IBM SPSS Statistics 20.0.

**Result:**

A total of 36 studies were included in the meta-analysis. Significant associations were found between liver fluke infection and cholangitis or cholecystitis (RR: 7.80, P<0.001; OR: 15.98, P<0.001), cholelithiasis (RR: 2.42, P = 0.03; OR: 4.96, P = 0.03), hepatocellular carcinoma (OR: 4.69, P<0.001) and cholangiocarcinoma (RR: 10.43, P<0.001; OR: 4.37, P<0.001). In addition, heavier infection was significantly associated with higher incidence of hepatobiliary pathological changes (P<0.05). However, cirrhosis was not significantly associated with liver fluke infection (RR: 3.50, P = 0.06; OR: 5.79, P = 0.08). The statistical heterogeneity was significant, no significant difference was observed in the sensitivity analysis, and no publication bias was found.

**Conclusion:**

The meta-analysis found that liver fluke infection was significantly associated with cholangitis, cholecystitis, cholelithiasis, hepatocellular carcinoma and cholangiocarcinoma and that more severe infection was associated with higher incidence. However, the association between liver fluke infection and cirrhosis was not significant.

## Introduction

At present, more than 750 million people throughout the world are at risk for infection with liver flukes, with an endemic concentration in southeast Asia and the western Pacific region[1]. The most important liver fluke species include *Clonorchis sinensis*, *Fasciola* spp. and *Opisthorchis* spp.[2]. The infectious metacercarial cyst stage is found in the meat of fish and shrimp as well as on the surfaces of water plants[3]. Once ingested, the metacercaria excysts in the duodenum, and the juvenile worm ascends the biliary tract through the ampulla of Vater[3]. The metabolites and mechanical stimulation of the liver fluke result in proliferation and inflammation in the epithelia of the biliary tracts as well as fibrosis and even cholangiocarcinoma[2, 4]. In humans, early and light infections may be asymptomatic or mild and are usually neglected. Infection by a large number of worms results in serious inflammation and leads to biliary tract obstruction, bile flux block and icterus[4]. However, the long-lived flukes cause chronic inflammation, which may be severe[5]. During chronic infection resulting from protracted episodes of re-infection over time, hepatic cells around the biliary ducts become denaturalized and putrescent, resulting in hepatic tissue atrophy and hepatocirrhosis[4, 6]. According to Keiser and Utzinger’s study, the global burden of food-born trematodiasis is 665,332 (479,496–859,051) DALYs (disability-adjusted life years). Moreover, they reported that food-borne trematode infections are among the most neglected of the so-called neglected tropical diseases[7, 8]. The awareness of liver fluke infection as a public health problem is insufficient because this infection impacts millions of people with severe morbidity and continues to emerge and expand. The increased infection rate of liver flukes may be due to factors such as the improved transportation and distribution systems to bring these aquatic foods to local and international markets[2, 8]. For example, in China, the current clonorchiasis rate is three times higher than that in the past decade[9, 10]. Findings of studies investigating the association between liver fluke infection and various hepatobiliary pathological changes have not been consistent, and systematic reviews and meta-analyses exploring the association have been even more limited. The present paper is based on a systematic review from cross-regional cohort studies and case-control studies to investigate the association between liver fluke infection and hepatobiliary pathological changes. This study will provide a more objective and comprehensive conclusion on this subject.

## Materials and Methods

### Search strategy

The study was performed using PRISMA (Preferred Reporting Items for Systematic Reviews and Meta-Analyses)[11]. The PRISMA statement is available in the supplementary data (S1 Table). Relevant literature that reported an association between liver fluke infection and hepatobiliary pathological changes was identified and screened from databases, including PubMed, Cochrane, Clinical Evidence, Trip Database, Clinical Trials, Current Controlled Trials, Web of Science, the China National Knowledge Infrastructure (CNKI) database, and the Wanfang academic journal full-text database. The following Medical Subject Heading (MeSH) terms were used individually and in combination in the search: “Fasciola hepatica,” “Clonorchis sinensis,” “Opisthorchis,” “Case-Control Studies,” “Cohort Studies,” “Cross-Sectional Studies,” “Hepatobiliary pathological changes,” “Cholangitis,” “Cholecystitis,” “Cholelithiasis,” “Cirrhosis,” “Hepatocellular Carcinoma” and “Cholangiocarcinoma.” The literature search was not limited by language or geographical region. The references in all of the retrieved articles were reviewed to search for additional relevant studies.

### Criteria for inclusion and exclusion

The inclusion criteria were as follows: (1) published full text available; (2) an observational study (a cohort study or a case-control study); (3) sufficient data reported to calculate the odds ratio (OR) with its 95% confidence interval (CI); and (4) the diagnosis of liver fluke infection based on (a) microscopy of liver fluke eggs in stool samples; (b) detection of worm-specific antibodies in serum samples or worm-specific antigens in serum or stool samples; (c) skin test with an intradermal injection of diluted crude live fluke antigen in veronal-buffered saline[12]; (d) observation of liver fluke eggs or parasites from bile, gallstones or intramural stones; (e) detection of diffuse dilatation of intrahepatic bile ducts in abdominal computed tomography (CT) or cholangiography; (f) results of molecular techniques such as polymerase chain reaction (PCR); or (g) history of liver fluke infection that could be confirmed by medical records. Studies were excluded if they were (1) comments, congresses, abstracts, reviews, or editorials without raw data or control subjects or (2) studies that included fewer than 10 participants.

### Data extraction

The following information was independently extracted from all of the included studies: the name of the first author, publication year, country or geographical area, liver fluke species, diagnostic methods for liver fluke infection, sample size, the number of the exposure or outcome of interest for case-control or cohort studies, respectively, and the quality of each study.

### Quality assessment

The quality of all of the included studies was assessed using The Newcastle-Ottawa Scale (NOS) (S2 Table). This scale involves a “star system” in which a study is judged on three broad perspectives: the selection of the study groups, the comparability of the groups and the ascertainment of either the exposure or outcome of interest for case-control or cohort studies, respectively. Studies having more stars are considered to be of higher quality.

### Statistical analysis

Statistical heterogeneity among studies was calculated using the χ^2^ test, P values, and I^2^ statistics[13]. A random-effects model was used to estimate the overall relative risk (RR) or overall odds ratio (OR) when heterogeneity was significant (Q: P<0.1, or I^2^>50%); if the reverse was true, a fixed-effects model was used (Q: P>0.1, or I^2^>50%). The overall RRs and ORs and their 95% CIs were estimated (P<0.05 was considered significant), and forest plots were generated for each disease associated with liver fluke infection. A sensitivity analysis was conducted, and publication bias was evaluated using funnel plots[14]. Meta-regression analyses were generated to explore possible sources of heterogeneity (adjusted R^2^>50% and P<0.05 were considered significant.) [15, 16], such as geographical area, decade of publication, liver fluke species, diagnostic methods and study sample size. Linear trend analyses were performed to determine the relationship between infection intensity and incidences of hepatobiliary pathological changes. Risk estimates, tests of heterogeneity, sensitivity calculations and publication bias analyses were performed using the Review Manager software, version 5.3; meta-regression analysis was performed using the Stata software, version 11.0 (Stata Corporation, College Station, TX, USA); and linear trend analyses were performed using IBM SPSS Statistics 20.0.

## Results

### Study characteristics

**Fig 1:**
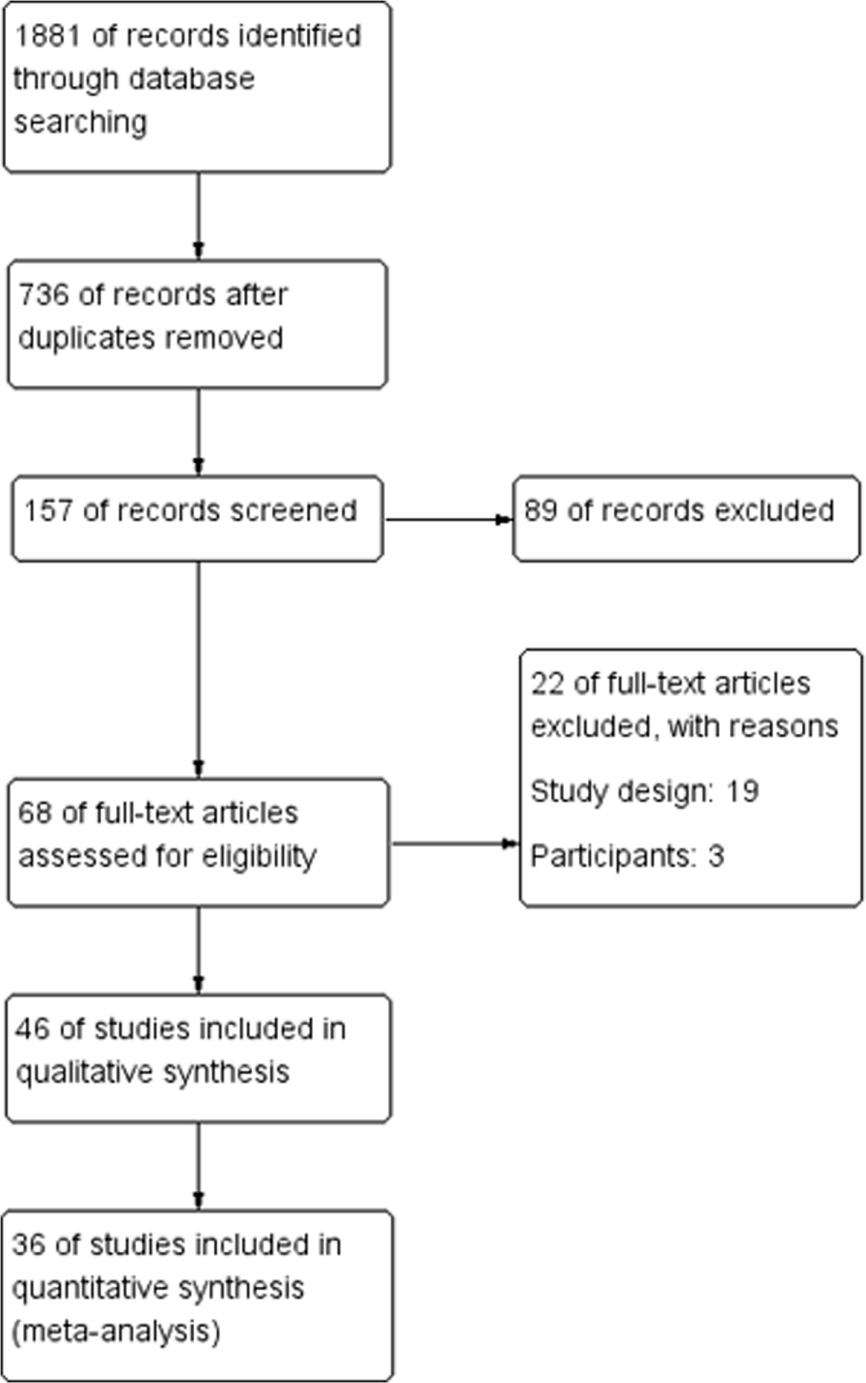
Flow chart of study selection.

A comprehensive search of databases provided 1881 potentially relevant citations, of which 10 cohort studies and 26 case-control studies met the study criteria and were included in the meta-analysis (Fig 1). Among the included studies, 14 were from mainland China[17–30], 1 was from Hong Kong[31], 2 were from Taiwan[32, 33], 7 were from Korea[34–40], and 11 were from Thailand[41–51]. The characteristics of the included studies with their quality are shown in Tables 1 and 2.

**Table 1:** Characteristics of included cohort studies on liver fluke infection and the risk of hepatobiliary pathological changes.

							Infected		Uninfected		Quality			
Pathological changes	Author	Year	Area	Liver fluke species	Diagnosis	Sample size	Events	Total	Events	Total	Selection	Comparability	Outcome	Reference
Cholangitis or Cholecystitis	Zhu SH	1982	China	*Clonorchis sinensis*	Pathologic examination	17603	381	2214	126	15389	★	★	★	[17]
	Mairiang E	1992	Thailand	*Opisthorchis viverrini*	Stool microscopy	95	27	71	4	24	★★★	★	★	[41]
	Chen ZZ	1997	China	*Clonorchis sinensis*	Stool microscopy	5230	79	1315	31	3915	★★★	★	★	[18]
Cholelithiasis	Zhu SH	1982	China	*Clonorchis sinensis*	Pathologic examination	17603	93	2214	46	15389	★	★	★	[17]
	Hou MF	1989	Taiwan	*Clonorchis sinensis*	Liver fluke history	1091	89	947	8	144	★★★	★	★★	[32]
	Mairiang E	1992	Thailand	*Opisthorchis viverrini*	Stool microscopy	95	6	71	0	24	★★★	★	★	[41]
	Choi MS	2005	China	*Clonorchis sinensis*	Stool microscopy	1384	279	1215	9	169	★★★	★	★	[19]
	Huang MH	2005	Taiwan	*Clonorchis sinensis*	Serologic test	131	12	47	46	84	★★★	★	★	[33]
	Kim HG	2009	Korea	*Clonorchis sinensis*	Several evidence lines	3080	45	396	340	2684	★★★	★	★	[34]
	Zhang X	2010	China	*Clonorchis sinensis*	Several evidence lines	1326	352	682	78	644	★★★	★	★	[20]
	Luo XB	2013	China	*Clonorchis sinensis*	Pathologic examination	340	49	153	30	187	★★★	★	★	[21]
Cirrhosis	Zhu SH	1982	China	*Clonorchis sinensis*	Pathologic examination	17603	128	2214	94	15389	★	★	★	[17]
	Huang MH	2005	Taiwan	*Clonorchis sinensis*	Serologic test	131	3	47	3	84	★★★	★	★	[33]
	Mairiang E	2012	Thailand	*Opisthorchis viverrini*	Stool microscopy	3359	182	404	656	2955	★★★	★	★★	[42]
Cholangiocarcinoma	Zhu SH	1982	China	*Clonorchis sinensis*	Pathologic examination	17603	5	2214	0	15389	★	★	★	[17]
	Mairiang E	1992	Thailand	*Opisthorchis viverrini*	Stool microscopy	95	2	71	0	24	★★★	★	★	[41]
	Huang MH	2005	Taiwan	*Clonorchis sinensis*	Serologic test	131	1	47	0	84	★★★	★	★	[33]

**Table 2:** Characteristics of included case-control studies on liver fluke infection and the risk of hepatobiliary pathological changes.

							Case		Control		Quality			
Pathological changes	Author	Year	Area	Liver fluke species	Diagnosis	Sample size	Exposure	Total	Exposure	Total	Selection	Comparability	Outcome	Reference
Cholangitis or Cholecystitis	Elkins DB	1990	Thailand	*Opisthorchis viverrini*	Stool microscopy	53	10	12	28	41	★★	★	★★	[43]
	Itoh M	1994	Thailand	*Opisthorchis viverrini*	Serologic test	69	29	49	0	20	★★★	★	★★	[44]
	Zheng ZX	1997	China	*Clonorchis sinensis*	Serologic test	53	6	14	1	39	★	★	★★	[22]
	Chen MF	2001	China	*Clonorchis sinensis*	Serologic test	117	12	38	1	79	★	★	★★	[23]
Cholelithiasis	Elkins DB	1990	Thailand	*Opisthorchis viverrini*	Stool microscopy	47	5	6	28	41	★★	★	★★	[43]
	Zheng ZX	1997	China	*Clonorchis sinensis*	Serologic test	53	6	14	1	39	★	★	★★	[22]
	Chen MF	2001	China	*Clonorchis sinensis*	Serologic test	117	12	38	1	79	★	★	★★	[23]
	Huang MH	2005	Taiwan	*Clonorchis sinensis*	Serologic test	252	9	131	1	121	★★	★	★★	[33]
	Choi D	2008	Korea	*Clonorchis sinensis*	Stool microscopy	134	3	67	1	67	★★★	★	★★	[35]
	Choi D	2008	Korea	*Clonorchis sinensis*	Serologic test	134	4	67	1	67	★★★	★★	★★	[35]
	Choi D	2008	Korea	*Clonorchis sinensis*	Radiological examination	134	10	67	16	67	★★★	★★	★★	[35]
Cirrhosis	Zheng ZX	1997	China	*Clonorchis sinensis*	Serologic test	49	2	10	1	39	★	★	★★	[22]
	Chen MF	2001	China	*Clonorchis sinensis*	Serologic test	129	12	50	1	79	★	★	★★	[23]
	Sripa B	2009	Thailand	*Opisthorchis viverrini*	Stool microscopy	328	46	200	20	128	★★★	★★	★★	[45]
Hepatocellular carcinoma	Chen HN	1994	China	*Clonorchis sinensis*	Liver fluke history	246	9	123	2	123	★★★	★★	★	[24]
	Shin HR	1996	Korea	*Clonorchis sinensis*	Stool microscopy	526	36	176	44	350	★★	★★	★★	[36]
	Shin HR	1996	Korea	*Clonorchis sinensis*	Liver fluke history	609	19	203	21	406	★★	★★	★	[36]
	Zheng ZX	1997	China	*Clonorchis sinensis*	Serologic test	111	16	72	1	39	★	★	★★	[22]
	Chen MF	2001	China	*Clonorchis sinensis*	Serologic test	98	4	19	1	79	★	★	★★	[23]
	Tan SK	2007	China	*Clonorchis sinensis*	Liver fluke history	1000	85	500	13	500	★	★	★	[25]
	Tan SK	2008	China	*Clonorchis sinensis*	Serologic test	944	73	444	12	500	★	★	★★	[26]
Cholangiocarcinoma	Gibson RB	1971	Hong Kong	*Clonorchis sinensis*	Stool microscopy	1401	11	17	310	1384	★	-	-	[31]
	Kim YI	1974	Korea	*Clonorchis sinensis*	Stool microscopy	1402	21	54	120	1348	★★	-	-	[37]
	Chung CS	1976	Korea	*Clonorchis sinensis*	Stool microscopy	595	19	36	88	559	★★	-	-	[38]
	Kurathong S	1985	Thailand	*Opisthorchis viverrini*	Stool microscopy	560	19	25	389	535	★★	-	-	[46]
	Elkins DB	1990	Thailand	*Opisthorchis viverrini*	Stool microscopy	49	8	8	28	41	★★	★	★★	[43]
	Parkin DM	1991	Thailand	*Opisthorchis viverrini*	Stool microscopy	202	43	101	9	101	★	★★	-	[47]
	Elkins H	1994	Thailand	*Opisthorchis viverrini*	Stool microscopy	1807	14	15	1383	1792	★★	-	-	[48]
	Itoh M	1994	Thailand	*Opisthorchis viverrini*	Serologic test	67	42	47	0	20	★★★	★	★★	[44]
	Shin HR	1996	Korea	*Clonorchis sinensis*	Stool microscopy	386	12	36	44	350	★★	★★	★★	[36]
	Shin HR	1996	Korea	*Clonorchis sinensis*	Liver fluke history	447	3	41	21	406	★★	★★	★	[36]
	Chen MF	2001	China	*Clonorchis sinensis*	Serologic test	85	3	6	1	79	★	★	★★	[23]
	Honjo S	2005	Thailand	*Opisthorchis viverrini*	Serologic test	253	65	126	8	127	★	★★	★★	[49]
	Choi D	2006	Korea	*Clonorchis sinensis*	Stool microscopy	244	3	122	5	122	★★	★★	★★	[39]
	Choi D	2006	Korea	*Clonorchis sinensis*	Pathologic examination	148	13	74	8	74	★★	★★	★★	[39]
	Choi D	2006	Korea	*Clonorchis sinensis*	Serologic test	328	25	164	11	164	★★	★★	★★	[39]
	Choi D	2006	Korea	*Clonorchis sinensis*	Skin test	276	19	138	12	138	★★	★★	★★	[39]
	Choi D	2006	Korea	*Clonorchis sinensis*	Radiological examination	370	156	185	57	185	★★	★★	★★	[39]
	Lee TY	2008	Korea	*Clonorchis sinensis*	Stool microscopy	2869	26	619	9	2250	★	★★	★★	[40]
	Poomphakwaen K	2009	Thailand	*Opisthorchis viverrini*	Stool microscopy	145	29	76	17	69	★★★	★★	★★	[50]
	Cai WK	2011	China	*Clonorchis sinensis*	*Not mentioned*	921	4	313	1	608	★	★★	★	[27]
	Peng NF	2011	China	*Clonorchis sinensis*	*Not mentioned*	294	18	98	19	196	★★	★★	★	[28]
	Wang XP	2012	China	*Clonorchis sinensis*	*Not mentioned*	302	6	102	3	200	★	★★	★	[29]
	Gao LB	2013	China	*Clonorchis sinensis*	Liver fluke history	640	2	128	2	512	★	★	★	[30]
	Manwong M	2013	Thailand	*Opisthorchis viverrini*	Serologic test	246	110	123	99	123	★★	★★	★★	[51]

### The risk of hepatobiliary pathological changes associated with liver fluke infection

#### Cholangitis or cholecystitis

**Fig 2:**
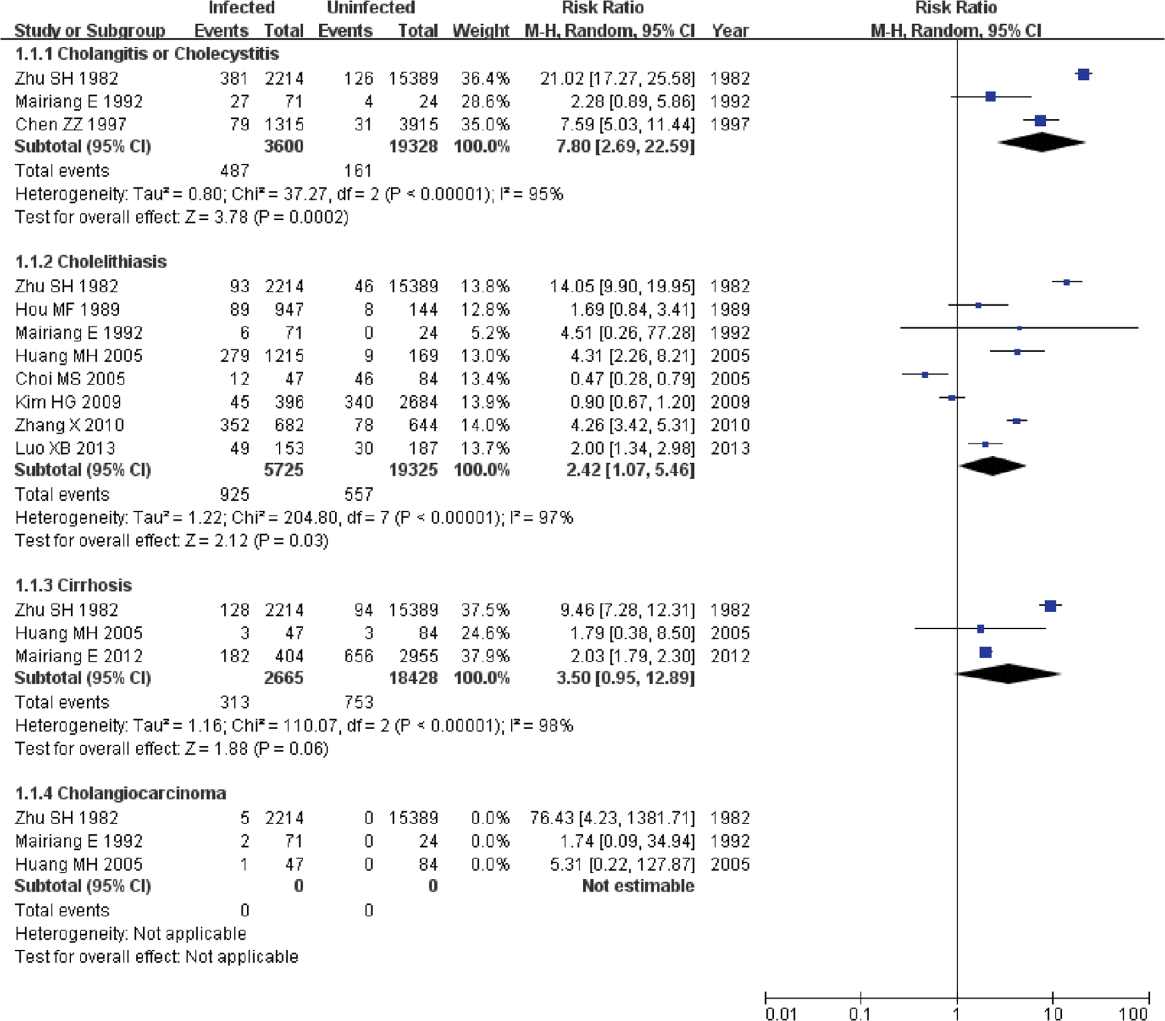
Forest plot of cohort studies on the relationship between liver fluke infection and various hepatobiliary pathological changes.

**Fig 3:**
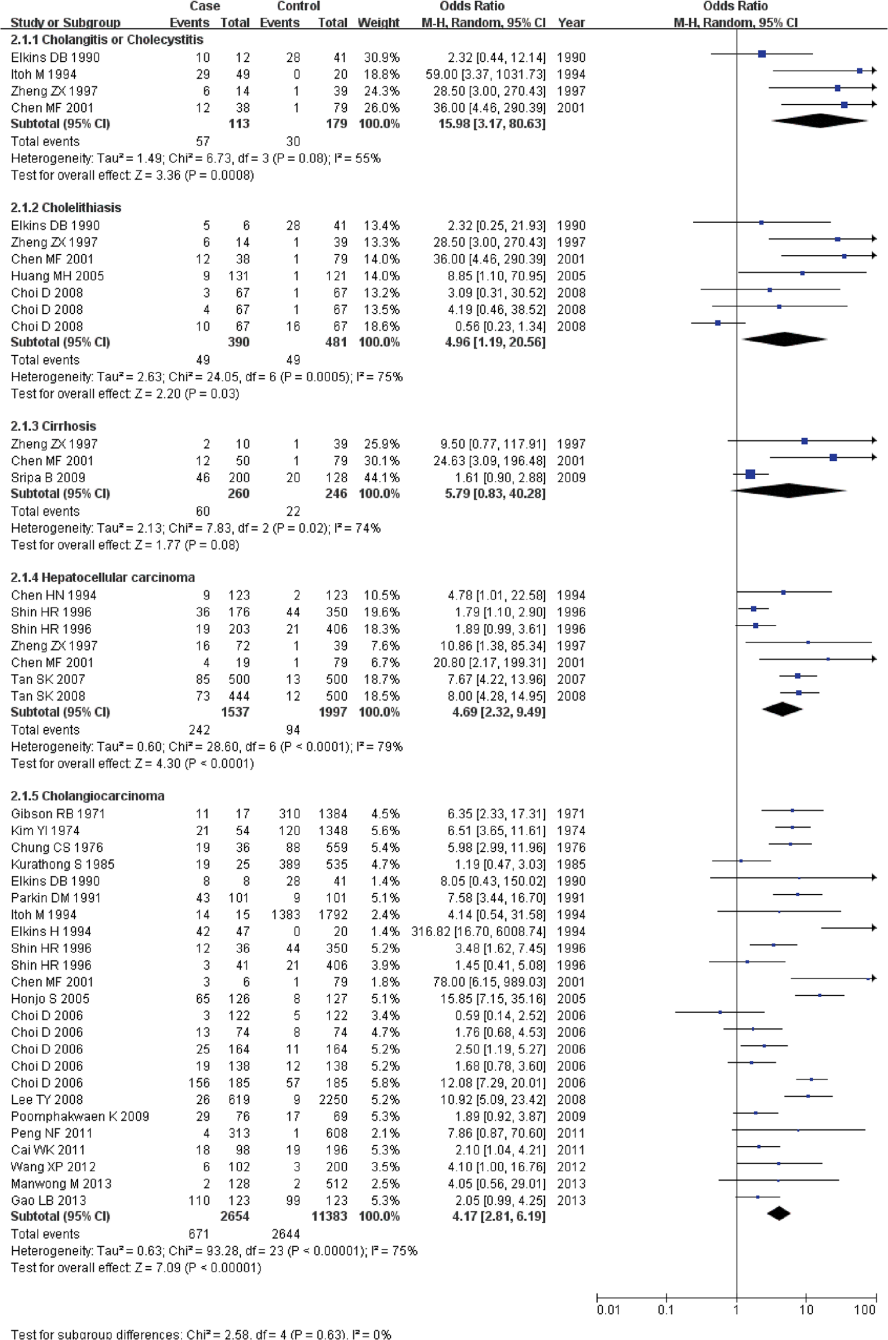
Forest plot of case-control studies on the relationship between liver fluke infection and various hepatobiliary pathological changes.

Several studies have reported a close association between liver fluke infection and cholangitis or cholecystitis [34, 52]. The overall RR with its 95% CI was extracted from the 3 included cohort studies[17, 18, 41], and the overall OR with its 95% CI was extracted from the 4 included case-control studies[22, 23, 43, 44]. The statistical heterogeneities of both the cohort studies and case-control studies were significant (I^2^ = 95%, P<0.001 and I^2^ = 55%, P = 0.08, respectively); hence, the overall RR for the cohort studies and the overall OR for the case-control studies were estimated using a random-effects model. The analysis of the cohort studies and case-control studies revealed that liver fluke infection was significantly associated with cholangitis and cholecystitis. (RR: 7.80, 95% CI: 2.69–22.59, P<0.001; OR: 15.98, 95% CI: 3.17–80.63, P<0.001) (Figs 2 and 3).

#### Cholelithiasis

Liver fluke infection has been investigated as a risk factor for cholelithiasis[21]. In total, 8 cohort studies[17, 19–21, 32–34, 41] and 5 case-control studies[22, 23, 33, 35, 43] were used to perform the respective meta-analyses using a random-effects model (I^2^ = 97%, P<0.001 and I^2^ = 75%, P<0.001, respectively). The analyses yielded an RR of 2.42 (95% CI: 1.07–5.46, P = 0.03) and an OR of 4.96 (95% CI: 1.19–20.56, P = 0.03), indicating that infection with liver flukes is a risk factor for cholelithiasis and that the association is significant (Figs 2 and 3).

#### Cirrhosis

In total, 3 cohort studies[17, 33, 42] and 3 case-control studies[22, 23, 45] on cirrhosis and liver fluke infection were identified and used to perform meta-analyses. A random-effects model was applied to the analyses (I^2^ = 98%, P<0.001 and I^2^ = 74%, P = 0.02, respectively). However, the result did not reveal a significant association between liver fluke infection and cirrhosis. For cohort studies, the overall RR of cirrhosis between infection with liver fluke and without infection was 3.50 (95% CI: 0.95–12.89, P = 0.06); for case-control studies, the overall OR of exposure to liver fluke infection between the case group and control group was 5.79 (95% CI: 0.83–40.28, P = 0.08) (Figs 2 and 3).

#### Hepatocellular carcinoma

Liver fluke infection has also been regarded as a risk factor for hepatocellular carcinoma [53]. Analysis of data from 6 case-control studies [22–26, 36] yielded inconsistent findings. The statistical heterogeneity was significant (I^2^ = 79%, P<0.001); thus, a random-effects model was applied. According to the analysis of the case-control studies, hepatocellular carcinoma was significantly associated with liver fluke infection with an OR of 4.69 (95% CI: 2.32–9.49, P<0.001) (Fig 3).

#### Cholangiocarcinoma

**Fig 4:**
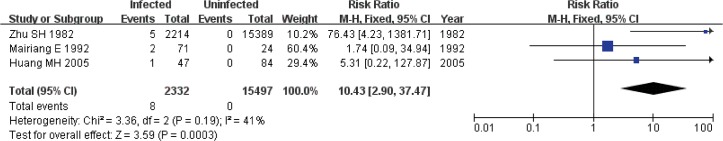
Forest plot of cohort studies on the relationship between liver fluke infection and cholangiocarcinoma.

The association between cholangiocarcinoma and liver fluke infection has been identified in articles over the last several decades [54, 55]. In our meta-analysis, 3 cohort studies[17, 33, 41] and 19 case-control studies[23, 27–31, 36–40, 43, 44, 46–51] were included. A fixed-effects model was used in the analysis of the cohort studies (I^2^ = 41%, P = 0.19), and a random-effects model was used (I^2^ = 77%, P<0.001) in the analysis of the case-control studies. The overall RR for the association between liver fluke infection and cholangiocarcinoma was 10.43 (95% CI: 2.90–37.47, P<0.001), and the association was significant. The overall OR for the association of cholangiocarcinoma with liver fluke infection was 4.37 (95% CI: 2.84–6.72, P<0.001), which indicated that liver fluke infection was a risk factor for cholangiocarcinoma (Figs 3 and 4).

### Sensitivity analysis

A sensitivity analysis was performed to identify whether the results of the meta-analysis were significantly affected by the exclusion of any individual study or the study with the highest quality or the greatest weight in the results. There was no significant impact observed in the overall ORs and 95% CIs.

### Publication bias

**Fig 5:**
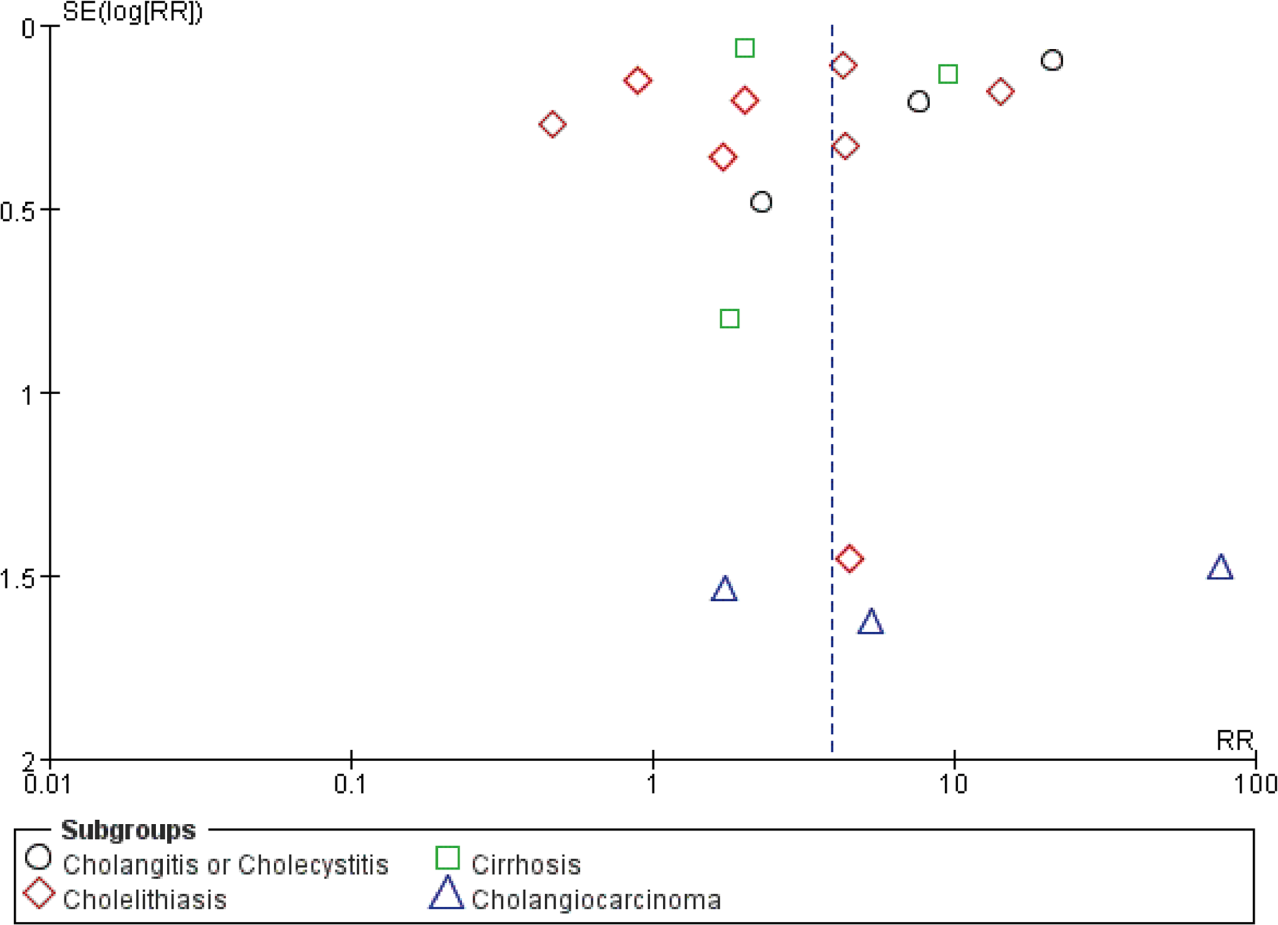
Funnel plot of cohort studies to detect publication bias.

**Fig 6:**
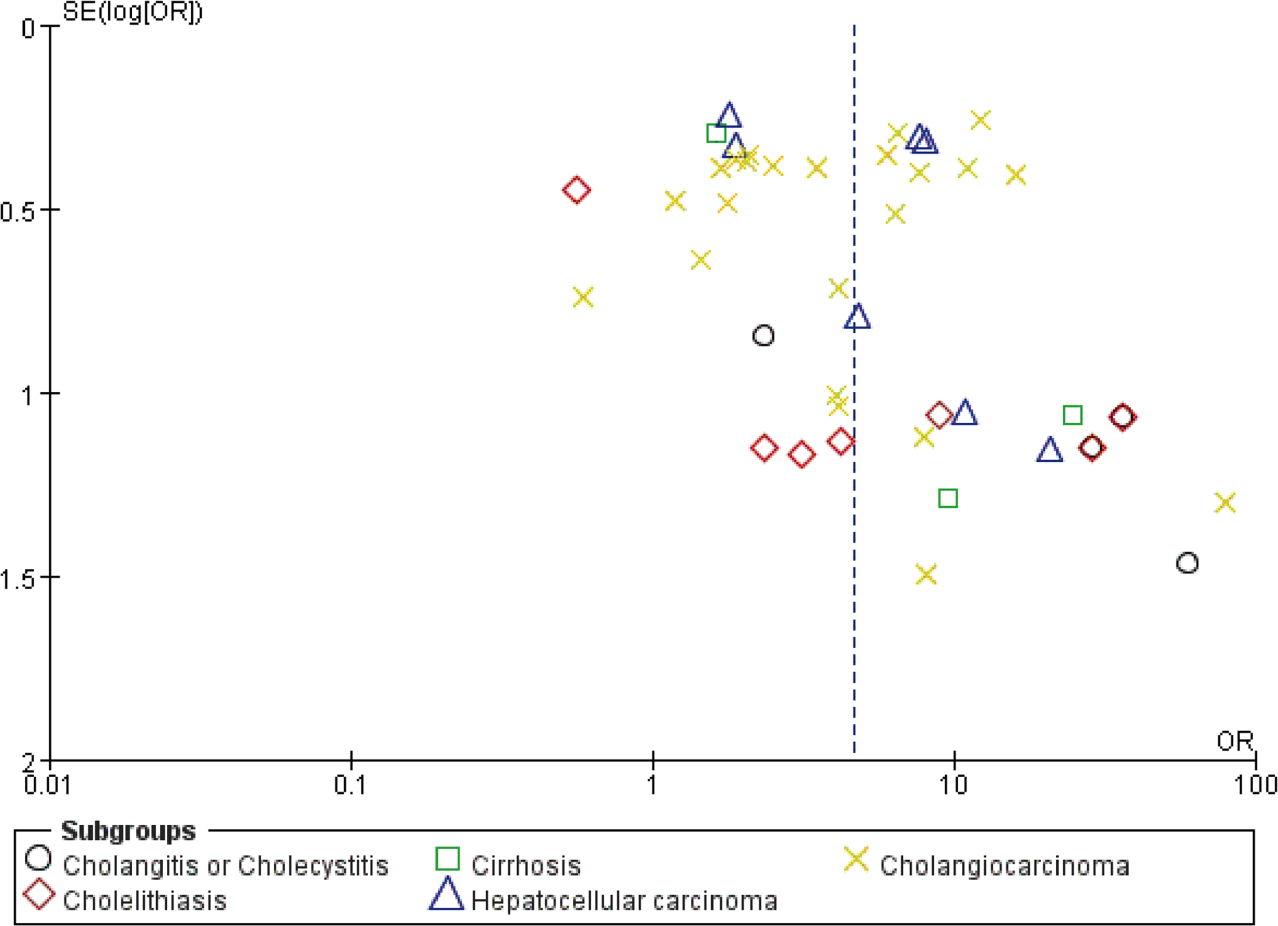
Funnel plot of case-control studies to detect publication bias.

Funnel plots of the studies in the meta-analysis were generated to evaluate publication bias (Figs 5 and 6). For both cohort studies and case-control studies, the plots approximately resembled a symmetrical funnel, and no publication bias was found.

### Meta-regression analyses

Meta-regression analyses were generated to explore possible sources of heterogeneity. Our meta-regression showed that geographical area, decade of publication, liver fluke species and diagnostic method did not contribute significantly to the heterogeneity (Adjusted R^2^<50% or P>0.05) for either cohort studies or case-control studies. In contrast, for cohort studies only, the study sample size did have a contribution (Adjusted R^2^ = 73.13%, P<0.001). The results of the meta-regression analyses are shown in Table 3.

**Table 3:** Results of the meta-regression analyses.

Study type	Factor	Adjusted R^2^	P
Cohort studies	Area	39.57%	0.009
	Decade of publication	28.85%	0.023
	Liver fluke species	-3.20%	0.475
	Diagnostic methods	32.05%	0.015
	Study sample size	73.13%	<0.001
Case-control studies	Area	10.92%	0.007
	Decade of publication	-2.46%	0.491
	Liver fluke species	-3.20%	0.705
	Diagnostic methods	-5.21%	0.822
	Study sample size	-6.80%	0.75

### Linear trend analyses of the dose-response relationship

In total, 2 cohort studies[19, 41] and 3 case-control studies[25, 43, 50] with intensity groups (≥3) of liver fluke infection were included in the linear trend analysis to examine the relationship between infection intensity and incidences of hepatobiliary pathological changes (Table 4). The results revealed a significant trend toward increasing incidences of hepatobiliary pathological changes with increasing intensity of liver fluke infection (P<0.05).

**Table 4:** Linear trend analysis.

								Test of linear trend		
Study type	Pathological changes	Author	Year	Infection intensity	Events	Total	Incidence	Value	Sig. (2-sided)	Reference
Cohort studies	Cholangitis or cholecystitis	Mairiang	1992	1 (EPG^a^ = 0)	4	20	20.0%	16.598	< 0.001	[41]
				2 (EPG = 200 to 1000)	4	16	25.0%			
				3 (EPG = 2000 to 7000)	11	16	68.8%			
				4 (EPG > 10000)	12	15	80.0%			
	Cholelithiasis	Mairiang	1992	1 (EPG = 0)	0	16	0.0%	4.983	0.026	[41]
				2 (EPG = 200 to 1000)	2	14	14.3%			
				3 (EPG = 2000 to 7000)	3	8	37.5%			
				4 (EPG > 10000)	1	4	25.0%			
		Choi	2005	1 (EPG = 0)	9	169	5.3%	150.063	< 0.001	[19]
				2 (EPG = 1 to 500)	54	532	10.2%			
				3 (EPG = 501 to 2000)	74	322	23.0%			
				4 (EPG ≥ 2001)	151	361	41.8%			
	Cholangiocarcinoma	Mairiang	1992	1 (EPG = 0)	0	16	0.0%	7.827	0.005	[41]
				2 (EPG = 200 to 1000)	0	12	0.0%			
				3 (EPG = 2000 to 7000)	0	5	0.0%			
				4 (EPG > 10000)	2	5	40.0%			
Case-control studies	Cholangitis or cholecystitis	Elikins	1990	1 (EPG = 0)	2	15	13.3%	5.321	0.021	[43]
				2 (EPG = 1 to 500)	2	14	14.3%			
				3 (EPG = 501 to 2500)	2	11	18.2%			
				4 (EPG = 2501 to 10000)	1	4	25.0%			
				5 (EPG > 10000)	5	9	55.6%			
	Cholelithiasis	Elikins	1990	1 (EPG = 0)	1	14	7.1%	4.711	0.03	[43]
				2 (EPG = 1 to 500)	1	13	7.7%			
				3 (EPG = 501 to 2500)	0	9	0.0%			
				4 (EPG = 2501 to 10000)	1	4	25.0%			
				5 (EPG > 10000)	3	7	42.9%			
	Hepatocellular carcinoma	Tan	2007	1 (Years^b^ = 0)	415	902	46.0%	57.423	< 0.001	[25]
				2 (Years < 10)	39	48	81.3%			
				3 (Years ≥ 10)	46	50	92.0%			
	Cholangiocarcinoma	Elikins	1990	1 (EPG = 0)	0	13	0.0%	12.306	< 0.001	[43]
				2 (EPG = 1 to 500)	0	12	0.0%			
				3 (EPG = 501 to 2500)	2	11	18.2%			
				4 (EPG = 2501 to 10000)	2	5	40.0%			
				5 (EPG > 10000)	4	8	50.0%			
		Poomphakwean	2009	1 (EPG = 0)	47	99	47.5%	4.353	0.037	[50]
				2 (EPG = 1 to 1000)	13	24	54.2%			
				3 (EPG > 1000)	16	22	72.7%			

## Discussion

Several published studies [52, 56, 57] have reported an association between liver fluke infection and various hepatobiliary pathological changes, including cholangitis, cholecystitis, cholelithiasis, cirrhosis, hepatocellular carcinoma and cholangiocarcinoma. However, these published studies have not identified consistent findings for the risk of these hepatobiliary pathological changes and liver fluke infection. In this systematic review and meta-analysis of cohort studies and case-control studies, significant associations were found between liver fluke infection and cholangitis or cholecystitis (RR: 7.80, 95% CI: 2.69–22.59, P<0.001; OR: 15.98, 95% CI: 3.17–80.63, P<0.001), cholelithiasis (RR: 2.42, 95% CI: 1.07–5.46, P = 0.03; OR: 4.96, 95% CI: 1.19–20.56, P = 0.03), hepatocellular carcinoma (OR: 4.69, 95% CI: 2.32–9.49, P<0.001) and cholangiocarcinoma (RR: 10.43, 95% CI: 2.90–37.47, P<0.001; OR: 4.37, 95% CI: 2.84–6.72, P<0.001). However, cirrhosis was not significantly associated with liver fluke infection (RR: 3.50, 95% CI: 0.95–12.89, P = 0.06; OR: 5.79, 95% CI: 0.83–40.28, P = 0.08). The observed statistical heterogeneity was significant, although sensitivity analysis did not alter the overall RR, overall OR, or their 95% CIs, and there was no evidence of publication bias.

A random-effects model was used in all of the analyses (except the analysis of cohort studies in cholangiocarcinoma) because significant heterogeneity was observed. Meta-regression analyses showed that the study sample size contributed significantly to the heterogeneity of the cohort studies (Adjusted R^2^ = 73.13%, P<0.001); as interpreted, the study sample size could explain 73.13% of the heterogeneity. In contrast, geographical area, decade of publication, liver fluke infection and diagnostic methods did not contribute to the heterogeneity. This result is most likely related to the limited information included in the studies, such as study design, the stages of pathological changes, and other demographic characteristics.

In this systematic review and meta-analysis, we found that liver fluke infection was significantly associated with increased risk of cholangitis and cholecystitis. The liver fluke secretes metabolites while invading, some of which are highly immunogenic, stimulating a strong humoral immune response that can be measured in the serum and bile[58]. Another study revealed that *Opisthorchis* antigens were observed along with an inflammatory cell infiltration, and the antigens were not only in the fluke itself but also in the biliary epithelium and surrounding tissue, which might then activate host immune responses[59].

Our study confirmed that liver fluke infection was significantly associated with cholelithiasis. The cause of clonorchiasis was most likely related to changes in the concentration of bilirubin, cholesterol, phospholipids, bile acid and the core of the gallstone formed from parasite debris or epithelial cells from the biliary ducts[60]. The metaplasia of bile duct epithelial cells into goblet cells and mucin secretion occurs in clonorchiasis and promotes a favorable environment for secondary bacterial infection[61].

A positive association was found between hepatocellular carcinoma and liver fluke infection. The mechanism of hepatocellular carcinoma in patients with liver fluke infection remains unknown. One possible mechanism is that epithelial ulceration and hyperplasia induced by the suckers of liver flukes induce stimulation of metabolites from the worms[62]. Secondary bacterial infection gives rise to periductal adenomatous hyperplasia and mucin secretion, which may result in hepatocellular carcinoma[62]. Another possible mechanism is that severin, a liver fluke excretory/secretory product, plays a key role in inhibiting apoptosis in human hepatocellular carcinoma cell lines and exacerbates hepatocellular carcinoma[63].

Our systematic review and meta-analysis confirm a significant relationship between infection with liver flukes and cholangiocarcinoma. The mechanisms by which liver flukes contribute to cholangiocarcinoma are multi-factorial[56] and include mechanical damage caused by the activities and movements of the worms, chronic inflammation, and the effects of parasite secretions[57].

This study confirms not only the relationship between liver fluke infection and various hepatobiliary pathological changes, such as cholangitis, cholecystitis, cholelithiasis, hepatocellular carcinoma and cholangiocarcinoma, but also the relationship between intensity of liver fluke infection and incidences of the hepatobiliary pathological changes. We found a significant trend toward increasing incidences of hepatobiliary pathological changes with increasing intensity of liver fluke infection. The ordinal intensity of liver fluke infection was analyzed by linear trend analyses instead of meta-analyses due to the limited sample size and the different ordinal scales used among the included studies. Additionally, information was too limited to generate analyses of the association between the intensity of liver fluke infection and the severity of pathological changes. However, in our included studies [41, 43], most cases of cholangiocarcinoma were identified from heavily infected patients, which supports the hypothesis that high pathogenicity relates to heavy parasite infection. The pathogenesis is due to the mechanical irritation by the flukes and some toxic substances produced by them[64].

Although published studies provided evidence to support the hypothesis that liver fluke infection is associated with cirrhosis [45], our analysis failed to provide sufficient evidence for this association. This inconsistency likely occurred because the studies that identified a relationship between cirrhosis and liver fluke were limited to animals, such as cattle, goats and sheep [65, 66]. In addition, most cirrhosis is associated with *Fasciola hepatica* infection [66, 67], which was not included in our analysis because of the absence of eligible studies.

Several limitations of our study deserve mention. First, non-English, non-Chinese studies were not included in our meta-analyses, which might have an impact on the overall results. Second, because of the limited number of studies involved and limited information on the studies, our study was not powered to perform subgroup analyses, which might provide reasons for the significant heterogeneity as well.

## Conclusion

In conclusion, our systematic review and meta-analysis found that liver fluke infection is associated with an increased risk of cholangitis, cholecystitis, cholelithiasis, hepatocellular carcinoma and cholangiocarcinoma, and more severe infection is associated with higher incidence. However, no significant evidence was found for the association between liver fluke infection and cirrhosis.

## Supporting Information

S1 Table PRISMA checklist for this meta-analysis. (DOC)

S2 Table The Newcastle-Ottawa Scale (NOS) for quality assessment. (DOC)
